# Radiological evolution of porcine neurocysticercosis after combined antiparasitic treatment with praziquantel and albendazole

**DOI:** 10.1371/journal.pntd.0005624

**Published:** 2017-06-02

**Authors:** Carla Cangalaya, Javier A. Bustos, Juan Calcina, Ana Vargas-Calla, Javier Mamani, Diego Suarez, Gianfranco Arroyo, Armando E. Gonzalez, Juan Chacaltana, Cristina Guerra-Giraldez, Siddhartha Mahanty, Theodore E. Nash, Héctor H. García, for the Cysticercosis Working Group in Peru

**Affiliations:** 1 Laboratorio de Inmunopatología en Neurocisticercosis, Facultad de Ciencias y Filosofía, Universidad Peruana Cayetano Heredia, Lima, Peru; 2 Facultad de Medicina Humana, Universidad Nacional Mayor de San Marcos, Lima, Peru; 3 Unidad de Cisticercosis, Instituto Nacional de Ciencias Neurológicas, Lima, Peru; 4 Facultad de Medicina Veterinaria, Universidad Nacional Mayor de San Marcos, Lima, Peru; 5 Facultad de Medicina Veterinaria y Zootecnia, Universidad Peruana Cayetano Heredia, Lima, Peru; 6 Facultad de Medicina, Universidad Peruana Cayetano Heredia, Lima, Peru; 7 Departamento de Diagnóstico por imágenes, Instituto Nacional de Ciencias Neurológicas, Lima, Peru; 8 Departamento de Ciencias Celulares y Moleculares, Facultad de Ciencias y Filosofía, Universidad Peruana Cayetano Heredia, Lima, Peru; 9 Laboratory of Parasitic Diseases, National Institute of Allergy and Infectious Diseases, National Institutes of Health, Bethesda, Maryland, United States of America; Michigan State University, UNITED STATES

## Abstract

**Background:**

The onset of anthelmintic treatment of neurocysticercosis (NCC) provokes an acute immune response of the host, which in human cases is associated with exacerbation of neurological symptoms. This inflammation can occur at the first days of therapy. So, changes in the brain cysts appearance may be detected by medical imaging. We evaluated radiological changes in the appearance of brain cysts (enhancement and size) on days two and five after the onset of antiparasitic treatment using naturally infected pigs as a model for human NCC.

**Methods and results:**

Contrast T1-weighted magnetic resonance imaging with gadolinium was performed before and after antiparasitic treatment. Eight NCC-infected pigs were treated with praziquantel plus albendazole and euthanized two (n = 4) and five (n = 4) days after treatment; another group of four infected pigs served as untreated controls. For each lesion, gadolinium enhancement intensity (GEI) and cyst volume were measured at baseline and after antiparasitic treatment. Volume and GEI quantification ratios (post/pre-treatment measures) were used to appraise the effect of treatment. Cysts from untreated pigs showed little variations between their basal and post treatment measures. At days 2 and 5 there were significant increases in GEI ratio compared with the untreated group (1.32 and 1.47 vs 1.01, p = 0.021 and p = 0.021). Cyst volume ratios were significantly lower at days 2 and 5 compared with the untreated group (0.60 and 0.22 vs 0.95, p = 0.04 and p = 0.02). Cysts with lower cyst volume ratios showed more marked post-treatment inflammation, loss of vesicular fluid and cyst wall wrinkling.

**Conclusion/Significance:**

A significant and drastic reduction of cyst size and increased pericystic enhancement occur in the initial days after antiparasitic treatment as an effect of acute perilesional immune response. These significant changes showed that early anthelmintic efficacy (day two) can be detected using magnetic resonance imaging.

## Author summary

Neurocysticercosis (NCC) is a frequent parasitic infection of the human brain and the most common cause of adult onset epilepsy in developing countries. Acute inflammatory response in NCC plays an important role in the pathogenesis of symptoms by anthelminitic therapies. The anthelmintic recommended therapy for NCC has drawbacks as the exacerbation of inflammation around degenerating cysts provokes the appearance of symptoms at the first days of treatment. Radiological changes in the appearance of cysts usually are seen after months of therapy. To evaluate if significant radiological changes (enhancement and size) occur in the first days of therapy, we used a porcine NCC model and magnetic resonance imaging (MRI) with contrast solution. The major radiological changes observed after treatment with albendazole and praziquantel were an increase in enhancement and the significant reduction in cyst size by day 2 and more evident on day 5. Cysts with greater changes also experienced exacerbated inflammation, loss of vesicular fluid and wrinkling of the cyst wall. These results show an early therapeutic effect and the possible utility of repeat MRI imaging within a few days after starting treatment. Finally, these findings contribute to our understanding of the treatment induced early exacerbation of symptoms.

## Introduction

Neurocysticercosis (NCC) is a neurological parasitic disease caused by the infection of the brain by the larval stage of *Taenia solium* [1]. NCC represents a serious and persisting public health problem because it is the most frequent cause of late-onset seizures in developing countries [1, 2].

Treatment with anthelmintic drugs such as praziquantel and/or albendazole has been associated with increased severity of symptoms within the first days of therapy [3–7]. Even though praziquantel and albendazole have different mechanisms of action [8, 9], both drugs cause the destruction of cysts and subsequent release of antigens, triggering the host immune response [7, 10–13]. Using the porcine NCC model and the antihelmintic drug praziquantel, this acute post-treatment inflammatory response was associated with pericystic inflammation [14] accompanied by an increase of vascular permeability, pro-inflammatory and regulatory cytokine profiles [15] during the second and fifth day. Using the same model, radiological changes in the appearance of brain cysts have been reported after two weeks of praziquantel treatment [16–18]. Similarly, the use of albendazole in the porcine model resulted in an increase of pro-inflammatory cytokines [14].

Medical imaging has been a useful tool in the diagnosis and medical follow-up of NCC patients [7]. Cyst appearance, size, perilesional enhancement and edema are imaging criteria to determine the radiological resolution of NCC after treatment [19]. The earliest radiological changes related to the size and appearances of brain cysts after conventional anthelmintic treatment has been reported during the first week of treatment in humans [20, 21] and in pigs after two weeks [16–18]. However, the radiological evolution of brain cysts during the first days of treatment, when perilesional inflammation establishes and symptoms increase in treated patients, has been scarcely explored.

In the present study, we evaluated the early radiological changes on MRI following the onset of antiparasitic treatment (days two and five) in pigs naturally infected with *T*. *solium* as a model for human NCC and confirmed the radiological findings with an ex-vivo histopathological examination.

## Materials and methods

### Study design and animals

A total of twelve pigs naturally infected with *Taenia solium* cysticercosis were obtained in endemic villages, transported to our facilities in Lima, and randomly divided in three groups, control or untreated, PZQ+ABZ 2d and PZQ+ABZ 5d, as follows: Four pigs remained untreated as a control group and 8 pigs were treated with the same combination of anthelmintic drugs and sacrificed at two (n = 4) and five (n = 4) days after treatment. The treatment consisted of combined therapy with praziquantel (Helmiben, Farmindustria, Peru) given for only the first day at 75 mg/kg/day, divided into three doses of 25 mg/kg administered every two hours [10], and albendazole (Zentel, GlaxoSmithKline, Peru) given daily until sacrifice at 15 mg/kg/day [22].

### Interventions

All pigs had pre and post-contrast MRI before treatment (Pre-treatment MRI) and on the day of sacrifice (Post-treatment MRI). Two hours before sacrifice, an Evans blue solution was infused as previously reported [15]. For all interventions, pigs were anesthetized with an intramuscular injection of a mixture of ketamine (Ket-A-100 50 mg/kg, Agrovet Market SA, Peru) and xylazine (Dormi-Xyl 2mg/kg, Agrovet Market SA, Peru) [15].

#### Image acquisition protocol

Pre- and post-treatment MRIs were performed on a 3-Tesla MRI scanner (Philips Achieva, Best, The Netherlands). Sequences included coronal, sagittal and axial TFE (Turbo field echo) T1-weighted gradient-echo images under conditions of repetition time (TR) = 7 miliseconds (ms), echo time (TE) = 4 ms, flip angle = 8°, pixel bandwidth = 270kHz, section thickness = 0.5–4 mm, matrix = 256–480 pixels, before and after injection of a contrast solution of gadolinium diethylene triaminopentaacetic acid (Gd-DTPA, 0.1 mmol/kg) through an ear IV catheter.

### Collection of specimens

After euthanasia, the pig brains were placed on dry ice slabs and cut in 1-cm sections. Cysts with pericystic capsules were collected from both hemispheres for histopathology and RNA studies. Specimens from the right hemisphere were fixed in 10% neutral buffered formalin, embedded in paraffin and then sectioned coronally at 4 μm thickness. Conventional hematoxylin-eosin was performed on every slide and two sections were examined with conventional light microscopy. Microphotographs were taken at 15X magnification with a Carl Zeiss stereoscope with AxioVision software to obtain a single large image (“cyst map”) [22].

### Radiological measures

#### Gadolinium enhancement intensity (GEI)

Quantitative measurements of gadolinium enhancement intensity (GEI) were obtained from pre- and post-treatment scan images, individually. The open access image-processing FIJI program (ImageJ, http://imagej.nih.gov/ij/) was used to adjust image brightness and contrast to better define and delineate individual pericystic lesion areas (selection of the region of interest). The gray values in each pixel within these selections were normalized as percentages of a continuous scale from 0 (black) to 256 (brightest) and an average GEI value from all pixels within the selection of each image/slice was calculated. The theoretical maximum value for GEI was 100%. Finally, using software R version 3.2.2, the average GEI from all section slices was obtained as a single value for each cyst [22]. These numerical values were ranged between 0 and 100. In addition, each cyst had two values of GEI from pre- and post-treatment scan images (preGEI and postGEI, respectively). Additionally, a ratio between pre (preGEI) and post treatment GEI (postGEI) was calculated to measure GEI increase after treatment for each cyst (postGEI/preGEI).

#### Cyst volume

Individual cyst volumes were calculated using coronal scan images based on spherical cap ([(1/6)*π*height*(3*area^2^+height^2^)]) and segment ([(1/6)*π*height*(3*(area_1_/π) + 3*(area_2_/π) + height^2^)]) formulas when cysts had three or more slice representations (S1 Fig). For cysts imaged in only one or two MRI slices, the ellipsoid volume formula (4/3* π*Long axis of sagittal*axial*coronal) was used. (Details of these calculations are provided in S1 Fig). Finally, we calculated the ratio between pre- and post-treatment cyst volumes to evaluate individual cyst volume reduction after treatment (postCyst Volume/preCyst Volume).

### Ex-vivo examination

#### Inflammatory score–composite (ISC)

The inflammation around the cyst (capsule) was measured using a semiquantitative histological score [14, 23, 24], which is described in a graphical manner in other studies [14]. Using this system, inflammatory stages (IS) are categorized as IS1 (a layer of collagen with scarce or no immune cells), IS2 (a thicker layer of collagen with an increased number of non-organized immune cells), IS3 (a typical granulomatous reaction with abundant organized immune cells distributed in layers [an epithelioid-rich cell layer next to the cyst wall, and increase of eosinophils] containing a few multinucleated giant cells [Langerhans cells]) or IS4 (with IS3 features plus additional abundant eosinophils distributed in a layer adjacent to the cyst wall and abundant multinucleated giant cells added to a severely damaged parasite structure). Different IS sectors may appear around the cyst perimeter, so we calculated the percentage of the cyst for each IS using a panoramic image of each cyst. The composite score is a weighted mean of the percentage of each inflammatory stage along the perimeter of a cyst, multiplied by the numerical value of the IS. [ISC = (% of IS1 * 1) + (% of IS2 * 2) + (% of IS3 * 3) + (% of IS4 * 4)]. Therefore the ISC results in a numerical variable with values between 100 and 400.

#### Cyst damage score–composite (CDSC)

Similar to ISC, we used a previously described scoring system to measure the cyst damage [14]. Cyst damage was categorized into four stages, CD0 (no damage), CD1 (few alterations of the cyst vesicular wall [tegument and subtegument layers] with overall preservation of the cyst architecture), CD2 (moderate alterations with loss of microtriches or microvilli on the outer tegumental layer, distended tegument and subtegument, and hyperchromophilic and dilated canalicular systems in the subtegument layer), or CD3 (severe alterations including loss of architecture of the vesicular wall and loss of definition in the internal region of the cyst) [14]. The composite damage score is a weighted mean of the extensions of each cyst damage stage along the perimeter of a cyst, multiplied by the numerical value of the CD [CDSC = (% of CD0 * 0) + (% of CD1 * 1) + (% of CD2 * 2) + (% of CD3 * 3)]. Values of the CDSC vary between 0 and 300 [14].

### Statistical analysis

Pre- and post-treatment GEI, pre- and post-treatment cyst volume, cyst volume ratio, GEI ratio, Inflammatory Score Composite (ISC) and cyst damage score composite (CDSC) were all continuous parameters. Treatments groups were used as a categorical variable (untreated, been treated at 2d and 5d). Mann Whitney test was used to compare pre-GEI and pre Cyst volume between the different treatment groups. Pre-post treatment differences for GEI and cyst volume were analyzed by the Wilcoxon test in each treatment group, individually. To evaluate if the mean change in GEI and cyst volume from pre to post-treatment measures differed in the three groups, we used a generalized estimating equation (GEE) analysis. To verify those post-treatment differences (cyst volume and GEI) truly result from treatment rather than from left-over effects of (usually random) pre-test differences between groups, we used an analysis of covariance (ANCOVA) with pre-treatment measures as covariates. Finally, we used the Mann-Whitney U test to compare ratios (changes between pre- and post-treatment measures) of GEI and cyst volume between treatment groups. Since ratio analysis results were highly correlated with unstandardized group analyses, we used ratios for the correlations with histopathology. Spearman correlation was used to assess the relation between each radiological (GEI and cyst volume) and histopathological (ISC and CDSC) parameters. All statistical analyses were performed using software R program for Windows, version 3.2.2. Graphs were performed using the ggplot2 package [25]. Values of p under 0.05 were considered to be statistically significant.

### Ethical statement

The study was conducted in accordance with the National Institutes of Health/AALC guidelines, and was reviewed and approved by the Institutional Ethics Committee for Animal Use at Universidad Peruana Cayetano Heredia (assurance number A5146-0).

## Results

### Experimental results

The study animals were seven male and five female pigs. Their weight range was variable (mean: 69.8 kg; range: 15–120 kg). A total of 328 brain cysts were obtained from the 12 pigs. The parasite cyst burden in each pig brain was also variable (mean: 27.3; range: 1–152) (Table 1) [22].

**Table 1:** General characteristics of study animals.

Pig characteristics	Treatment conditions		
	Control	PZQ+ABZ 2d	PZQ+ABZ 5d
**Number of pigs (male/female)**	4 (2/2)	4 (3/1)	4 (2/2)
**Weight (kg)**	120, 111, 78, 42	70, 45, 45,15	117, 90, 79, 25
**Total brain cysts (by pig)**	73 (1,11,16,45)	63 (10,11,13,29)	192 (4,13, 23,152)

### Gadolinium enhancement intensity (GEI)

A number of estimates of GEI showed increases around cysts in treated pigs compared to cysts in control untreated pigs. At baseline (before antiparasitic treatment), cysts in pigs from the Control and PZQ+ABZ 2d groups had higher GEI than cysts from the PZQ+ABZ 5d group (30.22 and 28.32 *vs*. 24.1, p<0.05). Post-treatment GEI values were higher in both treated groups compared with control pigs (PZQ+ABZ 2d: 36.04 and PZQ+ABZ 5d: 35.8 *vs*. Control: 33.31, p<0.001). When comparing pre- and post-treatment GEI in each group, there were marginal differences in cysts from control animals (30.22 vs. 33.31, p = 0.048), while GEI around cysts in treated groups increased markedly (PZQ+ABZ 2d: 28.32 *vs*. 36.04, p<0.001 and PZQ+ABZ 5d: 24.1 *vs*. 35.8, p<0.001) (S1 Table). GEE analysis confirmed that the effect of treatment in increasing the enhancement around cysts changed from basal to days 2 and 5 (RC for interaction term between pre-post GEI measures and groups: 4.996, <0.001) (S2 Table).

Additionally, after adjusting for pre-treatment differences, GEI increased significantly in both treated groups (PZQ+ABZ 2d: 7.324, p-value = 0.001 and PZQ+ABZ 5d: 9.442, p-value<0.001) compared with the control group (S2 Table).

**Fig 1:**
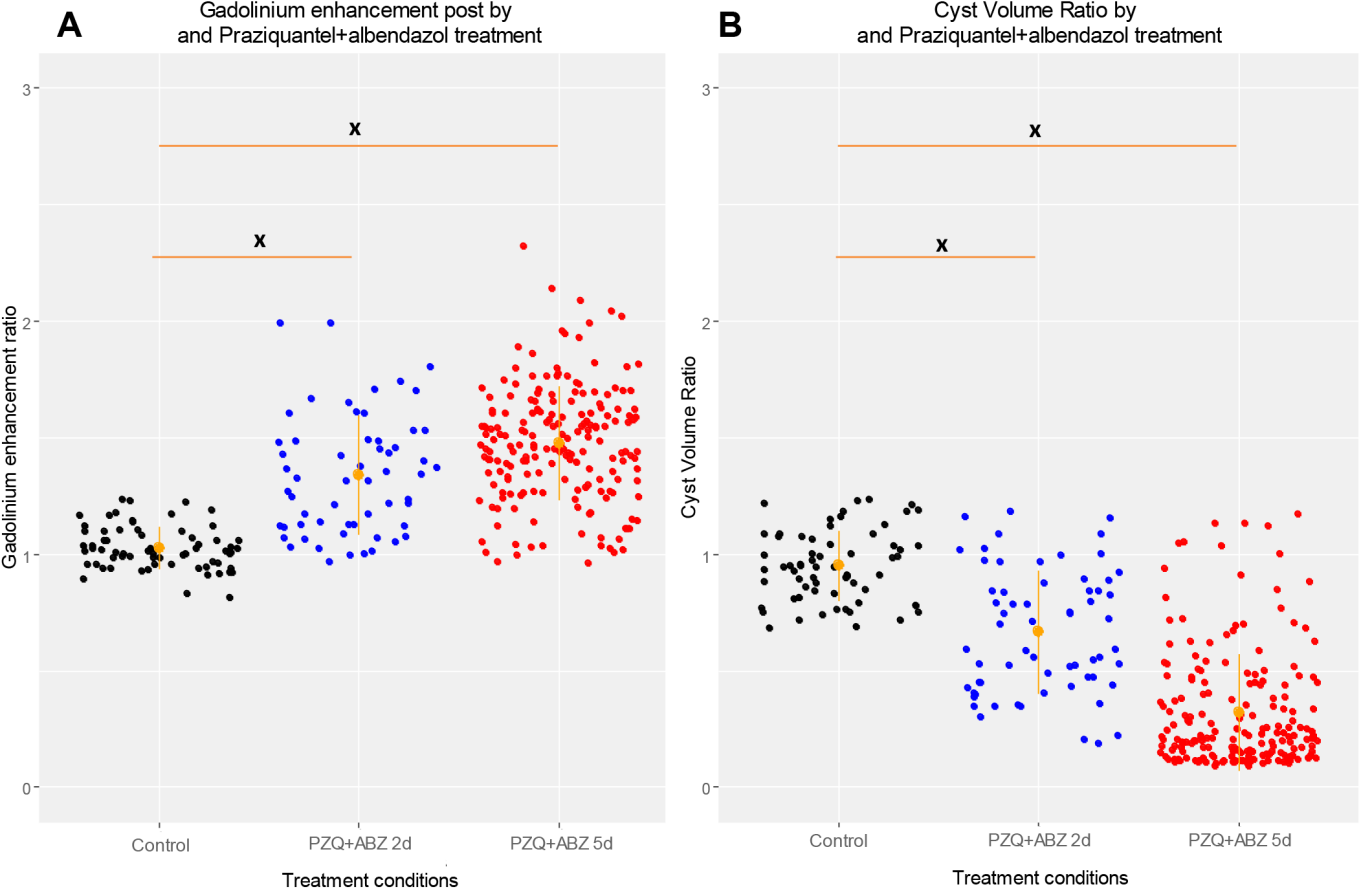
Imaging findings by treatment conditions. Dispersion graphs of post-treatment/baseline GEI Ratio (A, left) and Cyst Volume Ratio (B, right) by treatment groups, respectively. Orange lines show CI_95_ for the group. Asterisks indicate statistically significant differences in ratios between groups for Mann-Whitney test: *: p<0.05, **: p<0.01, ***: p<0.005, ****: p<0.001.

Ratio analysis was also used to assess the increases in enhancement between groups (across time). Individual cyst GEI ratio (post-/pre-treatment GEI) demonstrated a similar effect (mean ratios were 1.01 for cysts of control pigs, 1.32 for cysts of pigs in PZQ+ABZ 2d group, and 1.47 in PZQ+ABZ 5d group; p = 0.021 between groups and p = 0.387 comparing both treatment groups) (Table 2, Fig 1).

**Table 2:** Changes in contrast enhancement around cysts in pig brains as an effect of praziquantel plus albendazole treatment. Values represent medians and ranges of ratio GEI values of each brain cysts in each stratum.

Imaging results	Treatment conditions			*p-value C vs PA2d	*p-value C vs PA5d	*p-value PA2d vs PA5d
	Control Median (range)	PZQ+ABZ (PA)				
		PA2d Median (range)	PA5d Median (range)			
GEI ratio (postGEI/ preGEI)	1.01 (0.81–1.23)	1.32 (0.97–2.0)	1.47 (0.89–2.32)	0.021	0.021	0.387

* Mann Whitney test (adjusted per pig)

### Cyst volume

On baseline MRI (before antiparasitic treatment), cysts from the control and PZQ+ABZ 5d groups had larger volumes (106.16 mm^3^ and 114.18 mm^3^, respectively) than those from PZQ+ABZ 2d pigs (74.56 mm^3^) (p<0.05). On post-treatment MRI, cysts from PZQ+ABZ 2d and PZQ+ABZ 5d groups had lower cyst volume than cysts from the control group (48.64 mm^3^ *vs*. 97.92 mm^3^, <0.001 and 24.36 mm^3^ *vs*. 97.92 mm^3^, p = 0.03). Cyst volume also decreased in the 5-d treated cysts compared to the 2-d treated cysts (48.64 mm^3^ *vs*. 24.36 mm^3^, p<0.001) (S1 Table).

Similar to GEI, pre- and post-treatment cyst volumes in control pigs were similar (106.16 vs. 97.92, p = 0.045), while post-treatment cyst volumes were significantly smaller in treated animals (PZQ+ABZ 2d: 74.56 *vs*. 48.64, p<0.001 and PZQ+ABZ 5d: 114.18 *vs*. 24.36, p<0.001) (S1 Table). Adjustment for pre-treatment measures in ANCOVA confirmed that cysts from both treated groups had smaller volumes than cysts from the control group (PZQ+ABZ 2d: -62.117, p-value = 0.014 and PZQ+ABZ 5d: -95.032, p-value<0.001). Similar to enhancement, GEE analysis confirmed that the effect of treatment on cyst volume was more marked at day 5 (RC for interaction term between pre-post measures and groups: -48.201, <0.001) (S2 Table).

A similar effect was also seen when individual cyst volume ratios (post-/pre-treatment) were compared between groups. Cyst volume ratio was lower (more reduction) in cysts from both treated groups than in those from the control group (0.60 for ABZ+PZQ 2d and 0.22 for ABZ+PZQ 5d *vs*. 0.95 for controls, <0.05) (Fig 1), demonstrating cyst volume reduction after treatment. However, cysts from pigs in PZQ+ABZ 5d group had similar volume reduction than did cysts in the PZQ+ABZ 2d group (0.22 *vs*. 0.60, p = 0.248) (Table 3).

**Table 3:** Cyst volume variation as effect of praziquantel plus albendazol treatment. Values represent medians and ranges of post-/pre-treatment ratio of volume values of brain cysts in each stratum.

Imaging results	Treatment conditions			*p-value C vs PA 2d	*p-value C vs PA 5d	*p-value PA 2d vs PA 5d
	Control Median (Range)	PZQ+ABZ (PA)				
		2d Median (Range)	5d Median (Range)			
Cyst volume ratio (postVolume/preVolume)	0.95 (0.72–1.23)	0.60 (0.19–1.19)	0.22 (0.10–1.13)	0.04	0.021	0.248

* Mann Whitney test (adjusted per pig)

**Fig 2:**
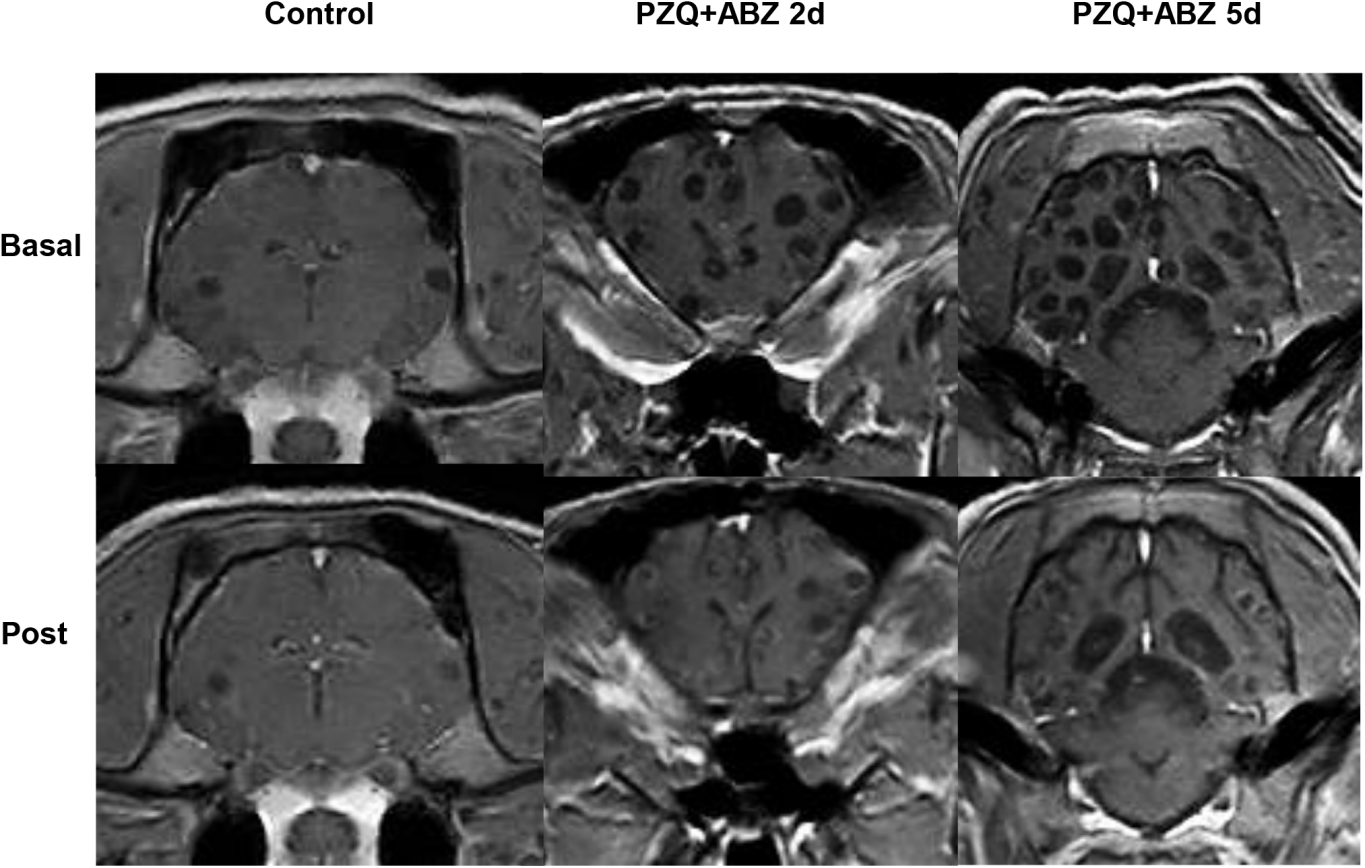
Radiological evolution after antiparasitic treatment with praziquantel and albendazole.

Further analysis demonstrated a significant negative relationship between cyst volume ratio with GEI ratio after 5 days of treatment (r = -0.412, p<0.001) (S3 Table, Fig 2) suggesting that cysts with more enhancement (GEI) experience greater reduction in volume.

### Imaging findings and histology

To confirm the radiological changes, we performed an ex-vivo examination to measure inflammation and the cyst damage using the ISC (inflammatory score-composite) and the CDSC (cyst damage score-composite), then we correlated those histological parameters with GEI and cyst volume ratios (radiological parameters).

Cysts from right brain hemispheres (n = 165) were selected for histopathological studies. Of these, only 105 cysts had a complete cyst structure and capsule and were therefore evaluable. Both treated groups had higher ISC and CDSC than the control group (p<0.001, Mann Whitney test). Both scores were higher at 5d compared to 2d, but there were no significant differences in these variables between both treated groups (ISC: 352 vs 304, p = 0.364; CDSC: 388 vs 336, p = 0.405 for CDSC) (S4 Table).

Higher ISCs were significant and positively associated with GEI ratio (r = 0.002, p = 0.028), meaning that cysts with higher increases in enhancement have more post-treatment pericystic inflammation. However, there was no significant correlation between GEI and CDSC (r = -0.001, p = 0.286) (S3 Table).

**Fig 3:**
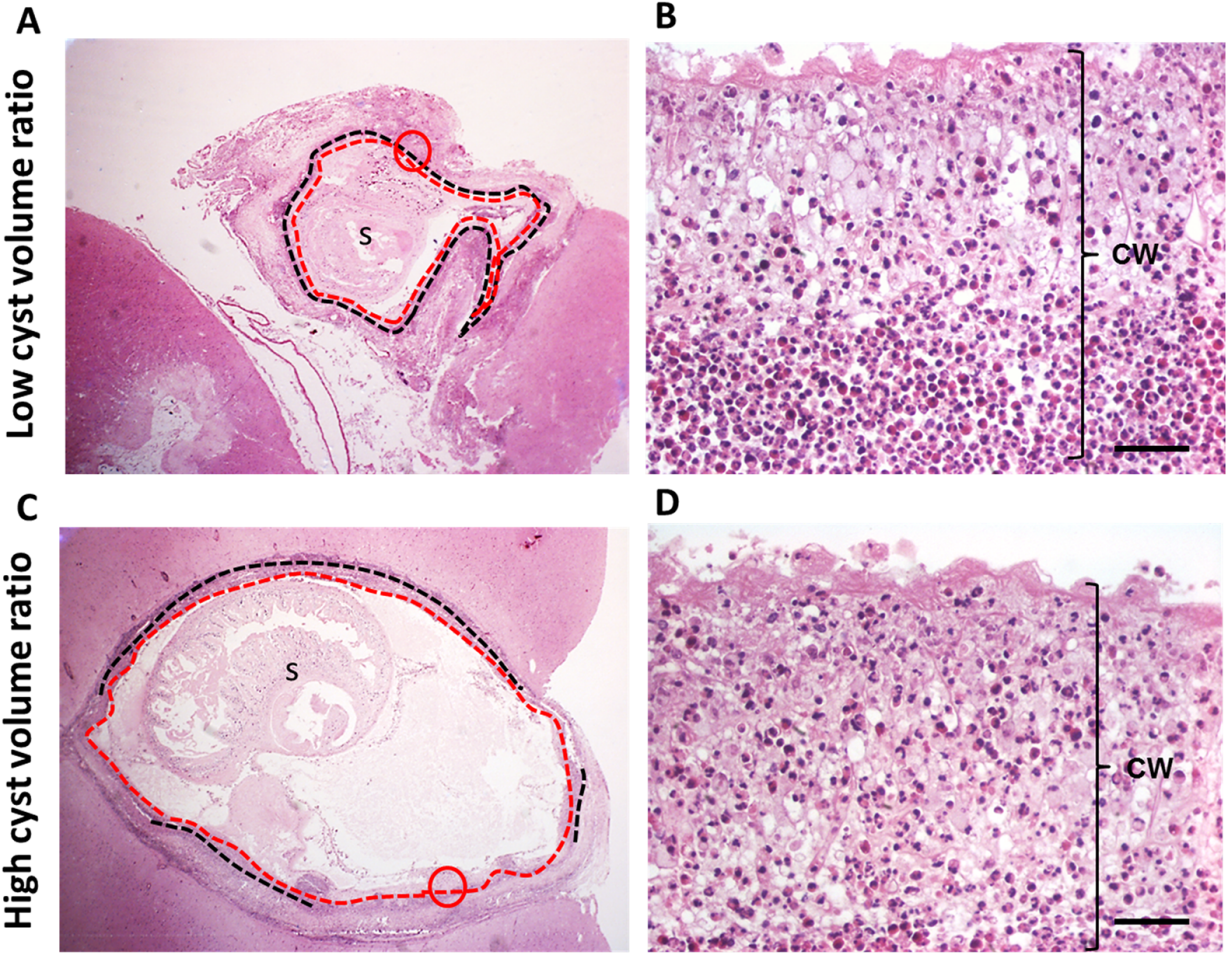
Histological changes and cyst volume variation at day 5. **A** and **B** shows two cysts from pigs of PZQ+ABZ 5d group. A representative cyst with high volume ratio (more cyst volume reduction on scan images) is seen in **A**, whereas a cyst with low volume ratio is shown in **B**. Black dotted lines indicate IS4 (higher stage of inflammation), red dotted lines delineate CD3 (higher stage of cyst damage), **s**: scolex and **cw**: cyst wall. Cysts in **A** and **C** had the same proportion of CD3 (100%), but **A** had more of IS4 (100%) than **C**. Thus, both cysts have the same CDSC (300) but different ISC (**A**: 400 and **C**: 360). **B** and **D** are the magnifications within the red circles in **A** and **C. B** and **D** show CD3, where the cyst wall is swollen with loss of its architecture and penetrated by eosinophils (bars = 50 um).

Interestingly, there was a significant negative relationship between cyst volume ratio (post-/pre-treatment measure) and post-treatment inflammation (ISC) at day 5 (RC = -0.002, p = 0.004), suggesting that cysts with increased inflammation showed increased reduction in volume. Slides of cysts with high volume ratio (higher reduction of cyst volume) showed loss of vesicular area and excess cyst wall folding upon themselves or wrinkling accompanied by granulomatous reaction (Fig 3A and 3C). In both cases, eosinophils have invaded the parasite’s wall as an effect of treatment (Fig 3B and 3D). This eosinophilic invasion has been observed before at points of high inflammation [26] and it is a demonstration of an acute response.

However, there was no significant relationship between volume ratio and cyst damage (CDSC) in any group (S3 Table).

## Discussion

Combined treatment of parenchymal NCC with praziquantel and albendazole destroys brain cysts in humans and pigs [1, 10], which is associated with a better clinical evolution in cases of human NCC [10]. However, after anthelmintic treatment humans are not usually reimaged until six or 12 months after treatment so early effects are not measured.

Despite the efficacy of combined treatment, in humans therapy causes an exacerbation of symptoms, usually seizures, due to acute inflammatory response to degenerating or dying cysts [27]. To assess early radiological changes, we examined MRI parameters of enhancement and cyst size and confirmed those findings with an ex-vivo histopathology (tissue-based semi-quantitative estimates of inflammation, and cyst damage) in naturally *T*. *solium*-infected pigs treated with albendazole and praziquantel at 2 and 5 days post initiation of treatment, compared to untreated control animals.

Enhancement has been associated to the disruption of the BBB in porcine NCC [22, 28, 29] as it happens in other diseases such as multiple sclerosis [30–32], gliomas, metastases and abscesses [33]. Earlier studies in NCC employing contrast-enhanced computed tomography (CT) in pigs [16–18] described the appearance of pericystic enhancement two weeks following praziquantel treatment. In humans, anthelmintic treatment also exacerbates gadolinium (Gd) enhancement during the first days of therapy [7], causing a change from an initial ring pattern of enhancement to a disc pattern, as seen using Gd T1-MRI [34]. These results are coherent with the post-treatment increase of enhancement reported in this study. We observed that the effect of treatment on enhancement increases with time already on day 2 and is further increased on day 5. Also, there was a positive correlation between increase of enhancement and inflammation. As enhancement is associated with BBB disruption, the following or parallel process that occurs is the extravasation of immune cells into the injured area and the increase of the inflammatory response. This agrees with previous studies where pro-inflammatory cytokines [14] and eosinophils where more abundant in pericystic tissues where the BBB had been disrupted [26].

Unexpectedly, we found that cyst volume was reduced very early after the onset of antiparasitic treatment. Reductions in cyst volume were evident in both treated groups on day two and were more pronounced five days after treatment, when the median of cyst volume loss was almost 78% ([1–0.22]*100; pre *vs*. post-treatment). Changes in the size of brain cysts in pigs had previously been reported after two weeks of praziquantel treatment [16]; however our findings suggest that sizable changes in the cyst size occur already by the second day of treatment. These results might have been more marked because we used combined therapy, and are consistent with early cyst size decrease observed on day 3 [21] and after one week [20] of antiparasitic treatment in humans. The reduction of the size of the parasite likely results from treatment-induced cyst damage and associated increased permeability of the cyst membrane, with a consequent increase in density of the cyst contents due to the influx of host albumin, protein coagulation, and loss of water [35].

The reduction of the size of the cyst was also accompanied with increased enhancement and inflammation. A previous study from our group reported that enhancement was associated with granuloma formation [22]; in this study we found similar results but additionally accompanied with cyst reduction. However, there was no association with cyst damage score (extension of the damage). A possible explanation could be that the combined treatment damages the scolex first, before damage is histologically noticeable and extended at the cyst wall level. Only afterwards would the cyst shrink and release fluid through the most heavily damaged regions of its wall. Similarly, a previous study concluded that the scolex is the primary target and its dissolution carries the complete resolution of the cyst [36]. As for cysts with little or no enhancement with a negligible change in size, they would represent those cysts in patients that do not respond to drug therapy, although our suggestion is valid only up to five days.

Despite these significant findings, our study has some limitations. We used a small number of animals and the parasite load per pig brain was very variable, making it difficult to compare groups. However, we used three statistical analyses to handle baseline differences to truly measure the effect of treatment. The variable thickness of MRI scans introduced some noise in the measurements of enhancement and volume; nevertheless, cyst volume and enhancement were significantly different in the treated groups. Minor drawbacks include use of only one hemisphere for histopathological assessments; however, differences in cyst load between hemispheres were not discernable [37]. Also, we used only two representative slides to assess the immune response of the entire cysts, which, nevertheless, sufficed to show differences in inflammation and cyst damage with treatment and over time. Despite these limitations, the changes observed after treatment corroborate the increase of inflammation seen in post mortem histological studies in pigs treated with antiparasitic drugs compared to untreated animals [14, 15] and were also confirmed by pre- and post-treatment MRI observations of gadolinium enhancement made in the same pigs. Finally, our study found that combined albendazole plus praziquantel treatment produces a rapid and pronounced reduction of the cyst size in the initial days of the treatment and an acute inflammatory response characterized by an increase of Gd enhancement. This may lead to a release of cyst contains by the extreme cyst damage and a subsequent reduction of cyst size. These results define the pathophysiology of the early exacerbation of symptoms induced by treatment of human NCC, which may lead to earlier monitoring of NCC treatment and thus improved and safer interventions.

## Supporting information

S1 Fig Calculation of cyst volume. **A** shows the areas calculated on the delineated objects from the scans. **B** shows the building of cyst volume based on the sum of the volumes of spherical caps 1 and 2 plus spherical segments 1, 2 and 3. **C** shows the formulas for spherical cap and spherical segment volumes.
(TIF)

S1 Table General values of imaging findings. p*: p-value of Wilcoxon paired test between pre- and post-treatment values in each stratum.
a: p-value of Mann-Whitney U test for comparisons of pre-treatment GEI and cyst volume values between control and treated groups.
b: p-value of Mann-Whitney U test for comparisons of post-treatment GEI and cyst volume values between control and treated groups.
(DOCX)

S2 Table ANCOVA and GEE analysis. RC: coefficient regression
(DOCX)

S3 Table Relationship between imaging and histopathology findings. *Univariate linear regression to model cyst volume ratio using GEI ratio, ICS and CDSC as covariates, and adjusted per pigs.
RC: coefficient regression.
(DOCX)

S4 Table Histopathological changes. Values represent medians and ranges of ISC and CDSC values of each brain cysts in each treatment conditions stratum.
*Comparisons between the control group and each treated group and between both treated groups were significant (p<0.001, Mann-Whitney U test).
(DOCX)
