# Supplementary figures and images for: Radiological evolution of porcine neurocysticercosis after combined antiparasitic treatment with praziquantel and albendazole
Source: PLoS Negl Trop Dis. 2017 Jun 2;11(6):e0005624. doi: 10.1371/journal.pntd.0005624 (PMC5470720; doi:10.1371/journal.pntd.0005624)

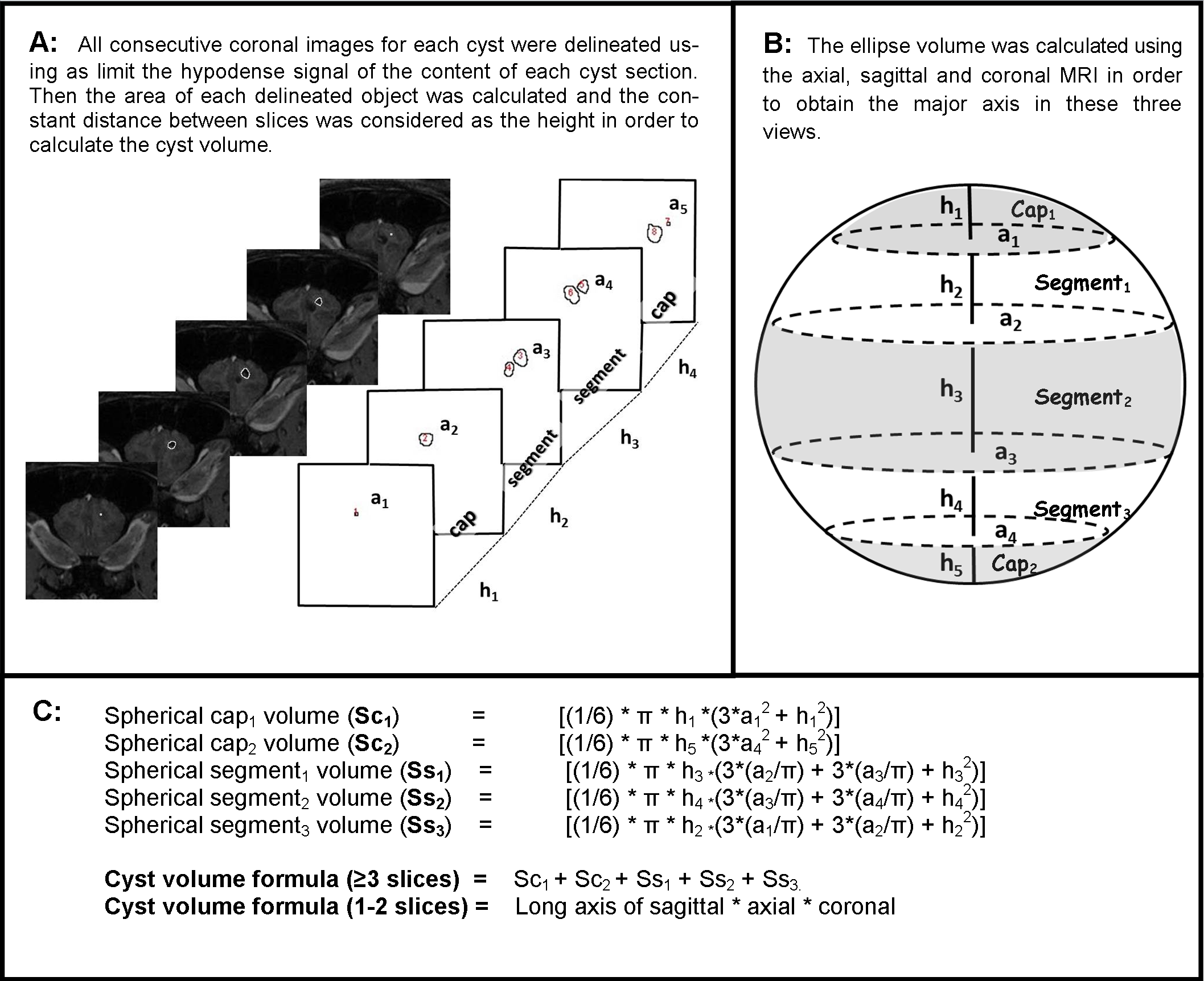

Supplement: S1 Fig — A shows the areas calculated on the delineated objects from the scans. B shows the building of cyst volume based on the sum of the volumes of spherical caps 1 and 2 plus spherical segments 1, 2 and 3. C shows the formulas for spherical cap and spherical segment volumes. (TIF) [file pntd.0005624.s001.tif]
