# Supplementary material for: Radiological evolution of porcine neurocysticercosis after combined antiparasitic treatment with praziquantel and albendazole
Source: PLoS Negl Trop Dis. 2017 Jun 2;11(6):e0005624. doi: 10.1371/journal.pntd.0005624 (PMC5470720; doi:10.1371/journal.pntd.0005624)
Supplement: S1 Table — p*: p-value of Wilcoxon paired test between pre- and post-treatment values in each stratum. a: p-value of Mann-Whitney U test for comparisons of pre-treatment GEI and cyst volume values between control and treated groups. b: p-value of Mann-Whitney U test for comparisons of post-treatment GEI and cyst volume values between control and treated groups. (DOCX) [file pntd.0005624.s002.docx]

**S1 Table. General values of imaging findings.**

|  | **Control** | | **PZQ+ABZ** | | | | **C vs PA2d** | **C vs PA5d** | **PA2d vs PA5d** |
| --- | --- | --- | --- | --- | --- | --- | --- | --- | --- |
|  |  |  | **2d** | | **5d** | |  |  |  |
|  | **PRE**  **Median (range)** | **POST**  **Median (range)** | **PRE**  **Median (range)** | **POST**  **Median (range)** | **PRE**  **Median (range)** | **POST**  **Median (range)** |  |  |  |
| **GEI** | 30.22  (16.13-43.65) | 33.31  (16.66-41.85) | 28.32  (16.55-44.2) | 36.04  (29.31-45.81) | 24.1  (11.66-33.13) | 35.8  (16.49-49.89) | 0.032^a^  <0.001 ^b^ | 0.001 ^a^  <0.001 ^b^ | 0.001 ^a^  <0.001 ^b^ |
| **p*** | 0.048 | | <0.001 | | <0.001 | |  | | |
| **Cyst volume**  **(mm^3^)** | 106.16  (17.39-1631.1) | 97.92  (21.85-2037.32) | 74.56  (15.47-593.84) | 48.64  (15.5-209.75) | 114.18  (30.23-686.92) | 24.36  (7.4-582.79) | 0.02 ^a^  <0.001 ^b^ | 0.31 ^a^  0.03 ^b^ | <0.001 ^a^  <0.001 ^b^ |
| **p*** | 0.045 | | <0.001 | | <0.001 | |  | | |

p*: p-value of Wilcoxon paired test between pre- and post-treatment values in each stratum.

a: p-value of Mann-Whitney U test for comparisons of pre-treatment GEI and cyst volume values between control and treated groups.

b: p-value of Mann-Whitney U test for comparisons of post-treatment GEI and cyst volume values between control and treated groups.
