# Supplementary material for: Radiological evolution of porcine neurocysticercosis after combined antiparasitic treatment with praziquantel and albendazole
Source: PLoS Negl Trop Dis. 2017 Jun 2;11(6):e0005624. doi: 10.1371/journal.pntd.0005624 (PMC5470720; doi:10.1371/journal.pntd.0005624)
Supplement: S2 Table — RC: coefficient regression (DOCX) [file pntd.0005624.s003.docx]

**S2 Table. ANCOVA and GEE analysis**

|  | **Enhancement**  **(RC, p-value)** | **Cyst volume**  **(RC, p-value)** |
| --- | --- | --- |
| **ANCOVA** |  |  |
| **Pre-treatment measure** | 0.818, <0.001 | 0.706, <0.001 |
| **Groups** |  |  |
| **PZQ+ABZ 2d** | 7.324, 0.001 | -62.117, 0.014 |
| **PZQ+ABZ 5d** | 9.442, <0.001 | -95.032, <0.001 |
| **GEE** |  |  |
| **Time (pre-post measures)** | 1.558, <0.001 | -1.39, 0.835 |
| **Groups** |  |  |
| **PZQ+ABZ 2d** | -0.505, 0.576 | -23.256, 0.380 |
| **PZQ+ABZ 5d** | -5.592, <0.001 | 9.388, 0.737 |
| **Interaction between groups and time** | 4.996, <0.001* | -48.201, <0.001* |

RC: coefficient regression. ANCOVA analysis was performed using General Linear model (GLM) adjusted by pig. After adjusting for pre-treatment measure, enhancement post-treatment increased by 7.324 and 9.442 points more in the PZQ+ABZ 2d and PZQ+ABZ 5d groups, respectively compared with the control group. Similarly, after adjusting for pre-treatment measure, post-treatment cyst volume decreased by 62.117 and 95.032 points in the PZQ+ABZ 2d group and PZQ+ABZ 5d, respectively compared with the control group. GEE analysis was performed using an independent within-group correlation structure and adjusted by pig. The mean change in the enhancement from pre to post-treatment gradually increased from control to PZQ+ABZ 2d and PZQ+ABZ 5d (RC:4.996, p-value<0.001). Similarly to the enhancement, the differences between pre and post-treatment cyst volume increased from control to PZQ+ABZ 2d and PZQ+ABZ 5d (RC: -48.201, p-value<0.001). This means that the effect of treatment on enhancement and cyst volume increase from control group to day 2 and day 5.
