# Supplementary material for: Radiological evolution of porcine neurocysticercosis after combined antiparasitic treatment with praziquantel and albendazole
Source: PLoS Negl Trop Dis. 2017 Jun 2;11(6):e0005624. doi: 10.1371/journal.pntd.0005624 (PMC5470720; doi:10.1371/journal.pntd.0005624)
Supplement: S3 Table — *Univariate linear regression to model cyst volume ratio using GEI ratio, ICS and CDSC as covariates, and adjusted per pigs. RC: coefficient regression. (DOCX) [file pntd.0005624.s004.docx]

**S3 Table. Relationship between Imaging and histopathology findings.**

|  | **Control**  ***RC, p-value** | **PZQ+ABZ 2d**  ***RC, p-value** | **PZQ+ABZ 5d**  ***RC, p-value** |
| --- | --- | --- | --- |
| **GEI ratio and Cyst volume ratio** | -0.001, 0.987 | -0.064, 0.634 | -0.35, <0.001 |
| **GEI ratio and ISC** | -0.0004, 0.387 | 0.0001, 0.953 | 0.003, 0.028 |
| **GEI ratio and CDSC** | -0.0001, 0.717 | -0.001, 0.193 | -0.001, 0.286 |
| **Cyst volume ratio and ISC** | 0.002, 0.3 | 0.0004, 0.838 | -0.002, 0.004 |
| **Cyst volume ratio and CDSC** | 0.0002, 0.605 | 0.0003, 0.726 | 0.002, 0.2 |

*Univariate linear regression to model cyst volume ratio using GEI ratio, ICS and CDSC as covariates, and adjusted per pigs.

RC: coefficient regression
