# Supplementary material for: Radiological evolution of porcine neurocysticercosis after combined antiparasitic treatment with praziquantel and albendazole
Source: PLoS Negl Trop Dis. 2017 Jun 2;11(6):e0005624. doi: 10.1371/journal.pntd.0005624 (PMC5470720; doi:10.1371/journal.pntd.0005624)
Supplement: S4 Table — Values represent medians and ranges of ISC and CDSC values of each brain cysts in each treatment conditions stratum. *Comparisons between the control group and each treated group and between both treated groups were significant (p<0.001, Mann-Whitney U test). (DOCX) [file pntd.0005624.s005.docx]

**S4 Table. Histopathological changes.** Values represent medians and ranges of ISC and CDSC values of each brain cysts in each treatment conditions stratum.

| **Histopathological changes** | **Treatment conditions** | | | ***p-value C vs PA2d** | ***p-value C vs PA5d** | ***p-value PA2d vs PA5d** |
| --- | --- | --- | --- | --- | --- | --- |
|  | **Control** | **PZQ+ABZ 2d** | **PZQ+ABZ 5d** |  |  |  |
| Numbers of evaluated pigs (cysts) | 3 (20) | 4 (30) | 4 (55) |  |  |  |
| ISC | 244  (128 -342) | 304  (217 - 400) | 352  (170 - 400) | <0.001 | <0.001 | 0.181 |
| CDSC | 200  (0-400) | 336  (200-400) | 388  (200-400) | <0.001 | <0.001 | 0.154 |

*Comparisons between the control group and each treated group and between both treated groups were significant (p<0.001, Mann-Whitney U test).
